# Role of the Juncturae Tendinum in Preventing Radial Subluxation of the Extensor Communis Tendons after Ulnar Sagittal Band Rupture: A Cadaveric Study

**DOI:** 10.5402/2012/597681

**Published:** 2012-05-30

**Authors:** N. Greville Farrar, Amrit Kundra

**Affiliations:** ^1^Department of Medical and Social Care Education, University of Leicester, Maurice Shock Medical Sciences Building, P.O. Box 138, Leicester LE1 9HN, UK; ^2^The Royal College of Surgeons of Edinurgh, Edinburgh, Midlothian EH89DW, UK

## Abstract

*Background*. Radial subluxation of the extensor communis tendons at the metacarpophalangeal (MCP) joints is a rarely reportedvinjury. These injuries have proved difficult to reproduce in cadaveric studies and have a low biomechanical likelihood of occurrence due to the ulnar direction of pull of the extensor communis tendons. It has been suggested that the juncturae tendinum may have a stabilising role, preventing radial subluxation after ulnar sagittal band rupture; however this has not been established. *Methods*. 40 cadaveric digits were dissected to reveal the extensor mechanism around the MCP joints. The ulnar sagittal bands were released and then the juncturae tendinum divided, in stages, before observing for radial subluxation or dislocation during finger flexion. *Results*. Radial subluxation of the extensor tendon was observed in only one digit after complete ulnar sagisttal band release. When all the fingers were flexed, after the juncturae tendinorum were divided, four additional tendons subluxed radially and a fifth tendon dislocated in this direction. When the digits were then flexed individually, there were eight unstable tendons in total. *Conclusions*. The juncturae tendinum appear to have a role in stabilising the extensor communis tendons at the MCP joints and preventing radial subluxation after ulnar sagittal band rupture.

## 1. Background

Ulnar subluxation of the extensor communis tendons at the metacarpophalangeal (MCP) joints occurs chronically in the rheumatoid hand, for which surgical repair is a well-established technique [1]. Acute traumatic disruption also occurs and has become commonly termed as “boxer's knuckle”. Descriptions of the mechanism of tendon subluxation in boxer's knuckle have concentrated on damage to the radial sagittal bands, with ulnar subluxation at the MCP joints being the resultant deformity, and pain on forming a closed fist often the presenting complaint of patients.

Traumatic radial subluxation occurs infrequently, and only a few cases have been reported. It has occurred in boxers after repetitive punching trauma in five cases [2]. It has also been described as having occurred after a single well-executed karate punch, once after “flicking a spider,” and one congenital case has been reported [3–5].

Subluxation in the ulnar direction is found most commonly in Boxer's knuckle, and two cadaveric studies have confirmed the biomechanical likelihood of this occurrence [6, 7]. The ulnar direction of pull of the extensor communis tendon, from its origin at the lateral epicondyle of the humerus, is at its greatest at the index finger MCP joint and least at the small finger MCP joint. These biomechanical principles were mirrored in the results of a cadaveric study, with ulnar instability proving to be most pronounced at the index finger and least at the small finger [7]. When looking at radial subluxation after ulnar sagittal band rupture, it could therefore be anticipated that subluxation would be most likely to occur in the small finger extensor tendon, as the inhibiting ulnar pull is minimised. Radial subluxation did indeed occur in the small finger in 3 out of 5 cases in the only case series described [2].

The mechanism of radial subluxation is not fully understood and has only been investigated to a limited extent. Young and Rayan divided the ulnar sagittal bands in their cadaveric study but found that radial subluxation did not occur with physiological hand movements [7]. It has been suggested that the juncturae tendinum (intertendinous connections) may have a stabilising role, inhibiting radial subluxation after ulnar sagittal band rupture; however this was not tested [6].

This cadaveric study aimed to investigate the occurrence and mechanism of radial subluxation of the extensor tendons at the MCP joints, including the potential stabilising role of the juncturae tendinum.

## 2. Methods

10 formalin-preserved cadaveric hands were used in this study to provide 40 digits for dissection. 5 were right hands and 5 were left hands.

The passive range of motion of all the metacarpophalangeal joints was measured, using a hand-held goniometer, in order to provide comparison with living human digits. Without the availability of X-ray in the anatomy department we could not comment on subtle arthritic changes which could interfere with the MCP joint motion, but there were no gross arthritic changes noted in the specimens used.

**Figure 1:**
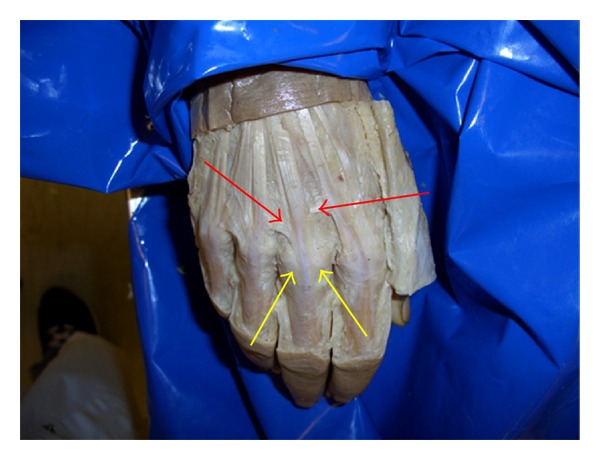
Sharp dissection to reveal the extensor tendons, the juncturae tendini (indicated by the red arrows), and the sagittal bands to the long finger (yellow arrows).

The dissection was kept to a minimum, in order to preserve the anatomical relationships. Skin and subcutaneous tissue was dissected on the dorsum only, from the level of the wrist joint to the distal interphalangeal joints. Sharp dissection was employed to carefully identify all the components of the extensor retinacular system, particularly the juncturae tendinum and the sagittal bands (see Figure 1).

For each digit, the position of the extensor tendon over the metacarpal head was noted throughout its normal range of motion. Subluxation and dislocation would be defined in accordance with previous definitions [8]. Subluxation is therefore lateral displacement with its border reaching beyond the midline, but remaining in contact with the condyle during full MCP joint flexion. Dislocation is defined as displacement of the tendon into the groove between the two metacarpal heads.

**Figure 2:**
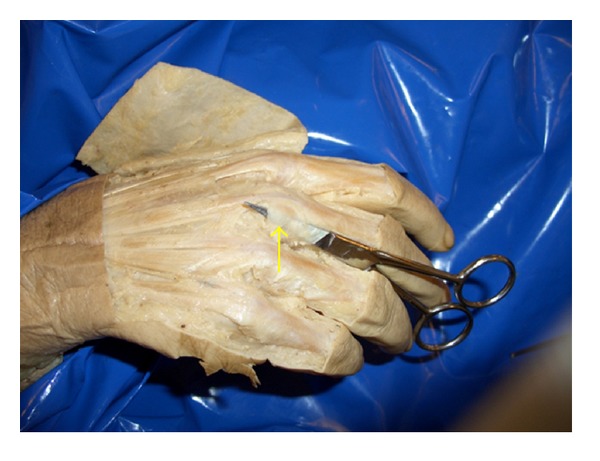
Identification of the ulnar sagittal band (indicated by the yellow arrow) prior to sectioning.

Continued dissection would be carried out in stages (see Figure 2). Initially the proximal half of the ulnar sagittal band would be divided using a scalpel. Subsequently, the whole ulnar sagittal band would be divided. Then, the juncturae tendinum would be divided in stages, starting with the most ulnar juncturae tendini through to the most radial.

For each stage of dissection, the metacarpophalangeal joint involved was taken through its full passive range of motion and the extensor tendon observed for subluxation or dislocation. This procedure was observed with the wrist in full extension, a neutral position, and then in full flexion. To simulate the functional position of fist formation, our experiments were carried out with all the metacarpophalangeal joints flexed together en masse. If no subluxation was observed, then the procedure would be repeated with the digits flexed individually.

## 3. Results

A hand-held goniometer was used to assess the passive range of motion of the cadaveric digits, and this is compared in Table 1 to a previous study of the active range of motion in healthy young volunteers [9].

The first stage of experimental dissection was to partially rupture the ulnar sagittal bands by dividing the proximal half with a scalpel. No extensor tendon instability occurred as a result of this manoeuvre. Subsequently, the ulnar sagittal bands were divided completely. Complete ulnar sagittal band release produced radial subluxation in only one digit. This was the long finger tendon and occurred in both fist formation and after individual flexion of the digit, but only with the wrist joint in a flexed position.

Table 2 summarises the occurrence of both tendon subluxation and dislocation after both ulnar sagittal band division and after subsequent division of the juncturae tendinum. All results for the table are for the wrist in a flexed position, as only one tendon was unstable with the wrist in the neutral position. This was a ring finger tendon, which would subluxate radially after ulnar sagittal band rupture alone.

With the wrist in the flexed position, only one tendon would subluxate radially, with the juncturae tendinum left intact. This was the same ring finger tendon that would subluxate with the wrist in the neutral position. The tendon was stable with the wrist in extension.

Once the juncturae tendinum were divided, four additional tendons subluxed radially and a fifth tendon dislocated in this direction. Four of the six unstable tendons were ring fingers, with one long finger and one index finger. When the digits were flexed individually, there were eight unstable tendons in total. One of the subluxating ring finger tendons dislocated after this manoeuvre, and two additional small finger tendons subluxated.

## 4. Discussion

Table 1 shows that the passive range of motion of the cadaveric digits was similar to the active range of motion, as tested in young adults [9]. The significance of this is that it was possible to assess for tendon subluxation over a physiological range of motion.

As can be seen in Table 2, only 1 out of 40 digits would subluxate radially after complete division of the ulnar sagittal bands. This was true for both combined flexion of all digits and individual finger flexion. No tendons would subluxate after partial, proximal division of the ulnar sagittal bands. These findings reinforce previous work, where radial subluxation was not reproduced after ulnar sagittal band rupture alone [7]. A low incidence of radial subluxation is also consistent with the ulnar bias exerted by the extensor digitorum communis tendons.

**Figure 3:**
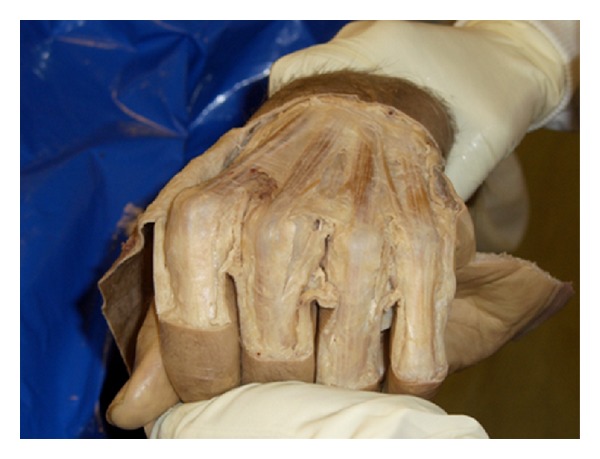
Radial subluxation of the long finger extensor communis tendon after complete ulnar sagittal band division and division of the corresponding ulnar juncturae tendinum.

Despite only 1 tendon subluxating radially after ulnar sagittal band division, further 5 tendons subluxed or dislocated after the additional division of the corresponding juncturae tendini.  Figure 3 demonstrates radial subluxation of the extensor communis tendon of the long finger after complete ulnar sagittal band division, plus division of the ulnar juncturae tendini. It can be seen that this tendon is unstable in the physiological position of flexion of all the digits at the MCP joints. This is the first time that radial subluxation has been reproduced in this position and lends support to the stabilising role of the juncturae tendinum.

A single case of radial subluxation has been reported after “flicking a spider” using the long finger, and this was investigated as part of the case report by the dissection of a single cadaveric digit [3]. Ulnar sagittal band division produced no subluxation when flexing all the digits; however, when the long finger alone was flexed, whilst resistance to flexion was applied to the other digits, the tendon would subluxate radially. This replicated the position of their patient's hand whilst “flicking a spider.”

We replicated the manoeuvre of flexing the digit in question, whilst resisting flexion of the other digits, and found that one of the subluxating tendons would now dislocate and two previously stable tendons would now subluxate radially. This provides further support to the hypothesis of Araki et al. that the resisted flexion of the other digits results in increased tension in the juncturae tendini and intertendinous fascia on the radial side of the affected digit [3]. This increased tension further destabilises the tendon towards the radial side. It seems therefore that the juncturae tendinum can act as stabilisers of the extensor communis tendons during coordinated flexion of the digits, but also as potential destabilisers during resisted flexion, whilst adjacent to a flexed digit.

All bar one tendon required the wrist to be in a flexed position before becoming unstable. These results are in agreement with previous studies [6, 7]. Young and Rayan used Swan-Ganz catheter measurements to test force generation in the sagittal bands and showed that pressure is maximal with the wrist in the flexed position, providing biomechanical evidence to support these findings.

Operative and nonoperative management of symptomatic ulnar subluxation after sagittal band injury in nonrheumatoid patients has been described in the literature [8, 10–12]. Watson et al. describe a successful technique using a distal extensor tendon slip on the side of the affected sagittal band to aid reconstruction, by looping it through the transverse metacarpal ligament. Rayan and Murray classified sagittal band injuries in 3 types, based on increasing severity, and suggested that surgery could be reserved for severe or chronic injuries, based on their successful results using buddy, or palmar splints.

With there being so few cases of radial subluxation reported, there is little precedent concerning management. Surgical repair has been reported as being successful with sagittal band repair alone [2] and after using an additional extensor tendon slip to aid repair in a case after a failed period of nonoperative management [4].

Juncturae tendinum reconstruction has been reported as a surgical management for ulnar tendon dislocation after injury without sagittal band damage [13]. In this case there was congenital absence of the juncturae tendinorum. It was unclear as to whether the dislocation was acute, as there was also tendon dislocation found on the uninjured side. Reconstruction was performed bilaterally and restored normal alignment and function.

In summary, we have provided some evidence in support of the role of the juncturae tendinum in stabilising the extensor communis tendons at the MCP joints. They may also have a potential destabilising role, depending on the finger positioning. Knowledge of their functional role may prove useful to surgeons when considering the operative stabilisation of unstable extensor tendons.

## Figures and Tables

**Table 1:** Range of motion of metacarpophalangeal joints (in degrees).

	Index		Long		Ring		Little	
	Flex (+)	Extend (−)	Flex (+)	Extend (−)	Flex (+)	Extend (−)	Flex (+)	Extend (−)
Cadaveric MCPs (*n* = 10)	92	12	94	16	95	16	97	19
Male MCPs (*n* = 60)	85	16	90	13	99	15	103	15
Female MCPs (*n* = 60)	86	26	90	13	99	15	103	15

**Table 2:** Tendon subluxations after complete ulnar sagittal band release and after subsequent division of the juncturae tendinum during fist formation and for individual finger flexion. (Note the figures in brackets are the combination of subluxations and dislocations occurring.)

		Index (*n* = 10)	Long (*n* = 10)	Ring (*n* = 10)	Little (*n* = 10)	All (*n* = 40)
Fist formation	Ulnar sagittal band rupture	0 (0)	0 (0)	1 (1)	0 (0)	1 (1)
	+ additional juncturae tendini division	1 (1)	1 (1)	3 (4)	0 (0)	5 (6)
Individual finger flexion	Ulnar sagittal band rupture	0 (0)	0 (0)	1 (1)	0 (0)	1 (1)
	+ additional juncturae tendini division	1 (1)	1 (1)	2 (4)	2 (2)	6 (8)
